# Meeting the WHO guideline on muscle-strengthening activities does not have to be time-consuming: a discussion of time-saving strategies

**DOI:** 10.3389/fpubh.2026.1872337

**Published:** 2026-07-08

**Authors:** Julian Brummer, Florian Herbolsheimer, Jackson Fyfe, Karen Steindorf

**Affiliations:** 1 Division of Physical Activity, Cancer Prevention and Survivorship, German Cancer Research Center (DKFZ), Heidelberg, Germany; 2 Medical Faculty, Heidelberg University, Heidelberg, Germany; 3 Institute for Physical Activity and Nutrition (IPAN), School of Exercise and Nutrition Sciences, Deakin University, Geelong, VIC, Australia

**Keywords:** exercise snack, guidelines, minimal-dose, muscle-strengthening activities, physical activity, resistance training, time

## Abstract

The World Health Organization (WHO) recommends performing muscle-strengthening activities at moderate or greater intensity, involving all major muscle groups, and on at least 2 days per week. However, estimates suggest only about 10–30% of the general population meet the guideline on muscle-strengthening activities, limiting overall (aerobic and muscle-strengthening) physical activity guideline adherence. Lack of time is a commonly cited barrier to being physically active and to performing muscle-strengthening activities specifically. To address this barrier, this Perspective describes evidence-based strategies, including exercise snacks, minimal-dose approaches, and time-saving programming (e.g., using supersets), that improve the time-efficiency of resistance training and may improve adherence to guidelines on muscle-strengthening activities. Upon introducing these strategies and the corresponding evidence, we describe how they could be applied in practice in various exemplary real-world scenarios differing in both time and equipment availability.

## 1 Introduction

Physical activity has a major positive impact on human health, well-being, and longevity (1–5). Public health bodies like the World Health Organization (WHO) advise engaging in a combination of moderate-to-vigorous-intensity aerobic physical activity (MVPA) and muscle-strengthening activities (MSAs) (1). Regarding the latter, the WHO advises adults to perform MSAs (1) involving all major muscle groups (2), at moderate or greater intensity, and (3) on at least 2 days per week (1).

Structured resistance training (RT) may be considered the primary form of MSA, and reliably induces muscular adaptations such as increases in strength and muscle size (6–10), which stand at the center of the WHO definition of MSAs (11). MSAs including structured RT are important for counteracting age-related declines in muscle mass, strength, and power (12). These activities also provide a range of additional health benefits (13–15), including improvements in risk factors for cardiovascular disease such as blood pressure and cardiorespiratory fitness (16–19), body composition (16, 20, 21), and musculoskeletal health (22, 23). These benefits are corroborated by additional epidemiological evidence indicating a protective effect of MSAs against various non-communicable diseases, including cardiovascular disease and certain cancers, as well as all-cause mortality (14, 15, 24–28). Importantly, at least some of the health benefits of MSAs seem to be independent of, and incremental to, the benefits provided by aerobic activities (14, 15, 29). Thus, the combination of both types of physical activity appears to be optimal (30), which supports their simultaneous inclusion in public health guidelines.

Despite the various health benefits, most people do not meet current MSAs guidelines. A recent meta-analysis found only around 23% of adults performed MSAs on at least 2 days/two times per week, as commonly recommended (31). This meta-analytic estimate aligns with related accounts reporting MSAs guideline adherence commonly ranges between 10 and 30% (14). Adherence to MVPA guidelines is usually much higher than adherence to MSA guidelines; as a result, MSAs typically represent the limiting factor for adherence to the combined MVPA *and* MSA guidelines (32–35), meaning that many people who fail to meet the combined guidelines do so because of insufficient MSA rather than insufficient MVPA. Increasing MSA guideline adherence would therefore also increase overall physical activity guideline adherence, which in turn would likely improve population health outcomes.

When examining population estimates of MSAs guideline adherence, consideration should also be given to the corresponding issues and limitations, though. Most importantly, most studies in the field relies on frequency-only assessments of MSAs (36, 37). Consequently, most insights in terms of MSAs guideline adherence are confined to whether people perform MSAs *often* enough, but not necessarily whether they also perform them at the recommended intensity and involve all major muscle groups. Especially considering whether all major muscle groups are being targeted has been found to further substantially reduce MSA guideline adherence (38). Taken together, the empirical evidence suggests MSA guideline adherence remains low, and improving current engagement in MSAs is an important avenue for disease prevention and health promotion.

While there is a clear need to improve engagement with MSAs, a recent systematic review concluded there is limited evidence on how to best increase the uptake and maintenance of MSAs (39). A lack of time is frequently reported as a major barrier to being physically active/exercising in general (40, 41) and also to engaging in MSAs/RT specifically (42, 43). To address this major barrier to engaging in MSAs, it seems particularly important to describe time-efficient ways of how MSAs guidelines could be met in practice, supported by related studies showing a high uptake and feasibility of short-duration physical activity and MSAs bouts (44, 45). Various previous articles have explored time-efficient RT programs (12, 46–49). However, to the best of our knowledge, no previous article has focused on collating strategies aimed at improving the ability of individuals to meet current MSAs guidelines and, in turn, meet overall physical activity guidelines. This is of great importance, though, as such an article could be used to update current physical activity guidelines and when interacting with public health communicators, policymakers, or the public directly.

Therefore, the present Perspective addresses this gap by translating the 2020 WHO recommendations on MSAs (1) into clear, time-efficient RT strategies that are compatible with everyday life considering varying time and equipment availabilities. To further enhance the practical applicability and extend previous research (50, 51), we also illustrate how these strategies could be used to meet the WHO guideline on MSAs in exemplary real-world scenarios.

## 2 Methods

For the present Perspective, which does not aim to provide an exhaustive overview of the literature, we performed a supportive literature search in the PubMed database (originally intended for a narrative review; registration available at https://osf.io/aw54c). The main search utilized the following search string: ((resistance[Title/Abstract] OR strength[Title/Abstract] OR weight[Title/Abstract] OR functional[Title/Abstract] OR circuit[Title/Abstract]) AND (training[Title/Abstract] OR exercise[Title/Abstract]) OR muscle-strengthening[Title/Abstract]) AND (time-saving[Title/Abstract] OR time-efficient[Title/Abstract] OR “time efficiency”[Title/Abstract] OR (exercise[Title/Abstract] AND snack*[Title/Abstract])).

Intervention studies in humans (primarily adults), or reviews/meta-analyses thereof, were of interest if at least one time-saving training strategy for RT as the core form of MSAs was investigated. Importantly, the present article does not constitute an exhaustive overview of all accounts provided in the scientific literature; rather, we present a selection of particularly suitable strategies that can be applied well to the general population. Therefore, we prioritized strategies utilizing conventional RT equipment (e.g., free weights, bodyweight) (16), as compared to those that necessitate costly and/or rare types of equipment (e.g., electromyostimulation). Additionally, we focused on strategies that can be applied well even outside of a gym or fitness center (e.g., only utilizing bodyweight exercises) or by individuals that are new to RT. Therefore, we do not detail strategies like dropsets (52) or blood flow restriction training (53, 54) that may be less applicable to beginners.

## 3 Time-efficient resistance training strategies

Based on the PubMed search yielding 634 articles and the identification of additional potentially relevant articles through the screening of reference lists and expert knowledge, it was evident that a range of time-saving strategies for MSAs have been discussed in the scientific literature. Following judgments of their applicability to the general population, including beginners, and the necessary equipment, four time-saving strategies were deemed particularly suitable for the purpose of the present Perspective: (1) minimal-dose approaches, (2) exercise snacks, (3) an appropriate exercise selection, and (4) supersets and circuit training. Table 1 provides a fully referenced summary of these strategies.

**Table 1:** Time-efficient resistance training strategies for meeting the WHO guideline on muscle-strengthening activities.

Strategy	Rationale/definition	Application	Supporting evidence
Minimal-dose approaches	Use the minimum RT dose that still induces favorable muscular adaptations.	Reducing the RT dose can be achieved by targeting different training variables, including frequency or volume (47). With respect to the WHO guideline on MSAs, a pragmatic minimal-dose approach could be to simply reduce MSAs frequency to the recommended minimum of 2 days per week (1). Training volume (e.g., defined as the number of sets per muscle group or specific exercise) constitutes one of the most important training variables, especially for inducing muscle hypertrophy (63), and can be subjected to minimal-dose approaches.	Performing RT or training a muscle on 2 days a week can induce measurable, near-optimal increases in muscle mass and strength (63). Low-volume RT programs can induce favorable muscular adaptations – even very minimalistic single-set RT programs (47). Even in resistance-trained individuals, such single-set RT routines performed twice weekly can induce muscle hypertrophy and lead to robust increases in muscle strength, power, and local endurance over the course of 8 weeks (64). Performing just a single set of 6 to 12 repetitions with a high intensity of effort (i.e., reaching volitional or momentary failure) 2 to 3 times per week can produce meaningful increases in maximal strength in the squat and bench press in resistance-trained men (48). The most comprehensive meta-analysis to date found a minimum effective dose for muscle hypertrophy and strength of four and one fractional weekly sets, respectively. This was based on the authors categorizing each RT set as direct or indirect based on its specificity to the hypertrophy or strength measurement enabling a more granular assessment of RT volume than previous analyses (63). For example, biceps curls vs. seated rows would have been exercises directly vs. indirectly targeting the biceps brachii, contributing one vs. a half “fractional” set, respectively (63).
Exercise snacks	Can be defined as “structured bouts of intense exercise dispersed across the day,” therefore strictly categorizing them as “exercise, not general lifestyle physical activity.” p.3 in (65)	Breaking up longer RT sessions into shorter sessions dispersed across the day, increasing time flexibility. Can be combined with other time-saving strategies, such as minimal-dose approaches.	While a lot of the work on exercise snacks and related concepts, such as vigorous intermittent lifestyle physical activity, has focused on aerobic activities (e.g., stairclimbing), various studies have incorporated MSAs snacks (44, 65, 66). Recent meta-analyses found beneficial effects of exercise snacks on maximal oxygen uptake, peak power output, total and LDL cholesterol (66) as well as muscle strength and endurance (67). The current body evidence on RT snacks as a subset of exercise snacks is limited due to the low number of studies as well as their designs, as they were mostly pilot or feasibility studies (45, 68–74), lacking long-term data. The existing data show particularly promising results regarding the feasibility and acceptability of RT snacks, though. An exemplary 28-day study in pre-frail older adults with a mean age of 78 ± 8 years found twice-daily, nine-minute RT snacks utilizing different bodyweight exercises (e.g., sit-to-stand from a chair, seated calf raises) to receive positive acceptability ratings and to be associated with potential benefits for physical function (68).
Exercise selection	Choose exercises that minimize the time required to perform them.	By performing multi-joint, bilateral compound exercises instead of single-joint, unilateral isolation exercises (e.g., bench press or push-ups instead of isolation exercises for the chest, shoulders, and triceps each), multiple major muscle groups and both sides of the body can be trained at once (46). By choosing exercises that require minimal or no time for set-up (e.g., using weight-stack machines or bodyweight exercises as compared to modalities like barbells that need to be loaded and unloaded), the overall time required to perform the exercises can be reduced even further (49).	Current evidence supports the use of time-efficient modalities like machines for effective RT (75, 76). A recent meta-analysis found that machine-based RT may even induce the greatest strength improvements among training modalities in older adults (77).
Supersets and circuit training	Modify how the single exercises and sets are performed within the training session to save time.	Supersets are a well-researched RT method, in which sets are alternated between two exercises with little or no rest between them, which is in contrast to traditional set structure, in which multiple sets of the same exercise are performed with interest rest periods before moving on to the next exercise (78). Circuit RT is an extension of superset RT, as it is characterized by repeating a sequence of exercises (more than two as with a superset) one after the other with minimal interset rest periods (79).	A recent meta-analysis found that supersets can induce muscular adaptations that are comparable to traditional set prescriptions, while reducing training time by around 37% (78). Various studies have shown that circuit RT can induce similar physiological adaptations as traditional RT, again while being much more time-efficient (79, 80). In addition, circuit RT might also be a potent stimulus for the cardiovascular system (81), making it a particularly interesting strategy for individuals who also struggle to meet the aerobic component of physical activity guidelines (1). Related strategies may also include training approaches like high-intensity functional training (e.g., CrossFit®), which are characterized by combining RT components with endurance-focused aspects (82).

MSAs, muscle-strengthening activities. RT, resistance training. WHO, World Health Organization.

Briefly, minimal-dose approaches are particularly suitable for reducing the number of training days per week (i.e., frequency), and the use of low-volume programs allows for a reduction in session duration by performing only few sets per muscle group/exercise. Given the diminishing returns in maximizing the muscular adaptations to RT, this supports the use of minimal-dose approaches in population groups that are less focused on achieving *optimal* muscular adaptations, but rather on feasible and time-efficient programs that provide the best “bang for buck.” Minimal-dose approaches can be well combined with exercise snacks, which can be used to break up longer RT sessions into shorter bouts that are more compatible with everyday life. Collectively, the findings of the studies conducted to date suggest that RT snacks are a well-accepted and time-efficient format for engaging in MSAs, especially among older adults. Evidence regarding beneficial muscular adaptations and improvements in physical function is emerging, but long-term data from randomized controlled trials are lacking. The time-efficiency of both minimal-dose approaches and exercise snacks can further be ensured by prioritizing multi-joint, bilateral compound exercises and exercises that require minimal or no time for set-up (e.g., using weight-stack machines as compared to barbells that need to be loaded and unloaded). Lastly, supersets and circuit training are exemplary time-saving strategies that do not alter the number of exercises or sets performed in an RT session but rather modify how the single exercises and sets are performed within the training session to save time, i.e., repeating multiple exercises one after the other with minimal interest rest periods.

## 4 Practical application

To illustrate the practical application of the time-saving strategies introduced above, Table 2 showcases how the strategies could be integrated into simplified RT prescriptions aligned with the WHO guideline on MSAs (1). In all three scenarios, minimal-dose approaches are used, some of which also make use of exercise snacks and/or additional time-saving programming strategies such as supersets. These imaginary scenarios span a range of time and equipment availabilities to increase their real-life applicability: Scenario 1 takes the perspective of a young university student whose schedule allows for two brief RT snacks (~five to ten minutes each) using bodyweight exercises and resistance bands every other day during the week, and no training during the weekend as their weekend days are strictly blocked for social events. In contrast, Scenario 2 applies to a middle-aged parent of two young children who is working full-time and whose schedule allows for one brief circuit training (~15 min) using bodyweight exercises and resistance bands during the week and one longer RT session (~30 min) in the gym. Scenario 3 features a retired older adult who prefers two longer sessions (~30 min each) in the gym each week. Here, it should also be mentioned that home-based RT options may be a particularly time-efficient setting for engaging in MSAs beyond applying time-saving RT strategies, as the additional travel time to and from a gym can be eliminated.

**Table 2:** Application of the selected time-saving strategies in three real-world scenarios for an exemplary week.

Scenario	Monday	Tuesday	Wednesday	Thursday	Friday	Saturday	Sunday
**Scenario 1:**^**a**^ Two brief RT snacks using bodyweight exercises and resistance bands on every other weekday	**Exercise snack 1 at home or in a park:** 2 sets of a superset consisting of a push-up variation (chest, triceps, shoulders) and a pull-up variation (back, biceps, shoulders) with 20 s rest in between exercises and 2 min rest in between supersets 1 set of a bicep curl with resistance bands (biceps) **Exercise snack 2 at home or in a park:** 2 sets of a superset consisting of a squat variation (legs) and a crunch variation (abdomen) with 20 s rest in between exercises and 2 min rest in between supersets	Free	**Exercise snack 1 at home or in a park:** 2 sets of a superset consisting of a push-up variation (chest, triceps, shoulders) and a bent-over row with resistance bands (back, biceps, shoulders) with 20 s rest in between exercises and 2 min rest in between supersets 1 set of a triceps extension with resistance bands (triceps) **Exercise snack 2 at home or in a park:** 2 sets of a superset consisting of a lunge variation (legs) and a Nordic hamstring curl variation (legs) with 20 s rest in between exercises and 2 min rest in between supersets	Free	**Exercise snack 1 at home or in a park:** 2 sets of a superset consisting of a push-up variation (chest, triceps, shoulders) and a bent-over row with resistance bands (back, biceps, shoulders) with 20 s rest in between exercises and 2 min rest in between supersets 1 set of lateral raises with a resistance band (shoulders) **Exercise snack 2 at home or in a park:** Exercise snack 1 at home or in a park: 2 sets of a superset consisting of a squat variation (legs) and a leg raise variation (abdomen) with 20 s rest in between exercises and 2 min rest in between supersets	Free	Free
**Scenario 2:**^**b**^ One brief circuit training using bodyweight exercises and resistance bands during the week and one longer session in the gym	Free	Free	**At home or in a park:** 3 circuits of the following exercises with 20 s rest in between exercises and 2 min rest in between circuits: Pull-up variation (back, biceps, shoulders) Push-up variation (chest, triceps, shoulders) Lunge variation (legs) Crunch variation (abdomen)	Free	Free	Free	**In a gym:** 2 sets of a superset consisting of a chest press (chest, triceps, shoulders) and a machine row (back, biceps, shoulders) with 20 s rest in between exercises and 2 min rest in between supersets 2 sets of a superset consisting of leg curls and leg extensions (legs) with 20 s rest in between exercises and 2 min rest in between supersets 2 sets of a leg raise variation (abdomen) with 2 min rest in between sets
**Scenario 3:**^**c**^ One gym session during the week and one gym session during the weekend	Free	Free	**In a gym:** 2 sets of a chest press (chest, triceps, shoulders) with 2 min rest in between sets 2 sets of a lat pulldown (back, biceps, shoulders) with 2 min rest in between sets 2 sets of a leg press (legs) with 2 min rest in between sets 2 sets of leg curls (legs) with 2 min rest in between sets 2 sets of a crunch variation (abdomen) with 2 min rest in between sets	Free	Free	Free	**In a gym:** 2 sets of hip extensions (legs) with 2 min rest in between sets 2 sets of leg extensions (legs) with 2 min rest in between sets 2 sets of a chest press (chest, triceps, shoulders) with 2 min rest in between sets 2 sets of a machine row (back, biceps, shoulders) with 2 min rest in between sets 2 sets of a leg raise variation (abdomen) with 2 min rest in between sets

All included exercises using free weights or machines can be adapted to a resistance that matches the individual’s capabilities, preferences, and goals. Similarly, exercise variations can be chosen that match the individual’s capabilities, preferences, and goals (e.g., a chest press machine, barbell bench press, dumbbell bench press, etc. would all classify as “chest press”). For bodyweight exercises, different variations can be applied to match an individual’s capabilities and preferences. In parentheses, it is specified which of the six major muscle groups named by the WHO (legs, back, abdomen, chest, shoulders, arms) (11) are the main contributors to the respective movement. The three exemplary scenarios were chosen to illustrate what different guideline-conforming MSAs programs could look like and are not meant to be exhaustive. MSAs, muscle-strengthening activities. RT, resistance training. WHO, World Health Organization.

a Scenario 1: A young university student whose schedule allows for two brief RT snacks (~five to ten minutes each) using bodyweight exercises and resistance bands every other way during the week, and no training during the weekend as their weekend days are strictly blocked for social events.

b Scenario 2: A middle-aged parent of two young children who is working full-time and whose schedule allows for one brief circuit training using bodyweight exercises and resistance bands during the week and one longer session in the gym when the children are with their grandparents. The circuit training is estimated to require about 15 min of time commitment. The gym session is estimated to require about 30 min of time commitment. While no direct exercises for the arms are incorporated, they are still involved in the compound movements for the chest and the back.

c Scenario 3: An older adult who is retired and whose schedule would allow for great flexibility, but due to personal preference, two roughly half-hour sessions in the gym are desired – one on Wednesday and one on Sunday. While no direct exercises for the arms are incorporated because the person does not enjoy them, they are involved in the compound movements for the chest and the back.

All included exercises using free weights or machines can be adapted to suit the individual’s capabilities, preferences, and goals, while for bodyweight exercises, different exercise variations can be applied. For example, incline push-ups with the hands placed on an elevated object can be used by less fit individuals, regular push-ups with the hands on the floor can be used by intermediates, and weighted or decline push-ups can be used by fitter individuals. Similarly, sit-to-stand variations could be performed by less fit individuals, while regular body weight squats may be more appropriate for intermediates, and pistol squats using only one leg at a time could be performed by fitter individuals.

## 5 Discussion

This Perspective aimed to describe pragmatic time-efficient strategies for meeting contemporary MSAs guidelines, which are currently met by only small proportions of the general population. A growing body of evidence supports the use of minimal-dose approaches, exercise snacks, and time-saving programming to create effective, yet time-efficient RT prescriptions, necessitating a weekly time commitment of only around 40 to 60 min. In addition to overcoming the barrier of having a lack of time, these proposed weekly durations of MSAs in the form of RT also align with the range of maximum health benefits established in two recent meta-analyses, for example pertaining to reductions in all-cause mortality (15, 25). Additionally, if the aim is to reduce the time commitment to an absolute minimum, the necessary time commitment could be reduced even further. For example, the single-joint isolation exercises for the arms and abdomen could be excluded given their involvement in the other multi-joint exercises. Even if only a subset of the proposed exercises can be performed, the available evidence suggests that engagement in *any* MSAs is expected to confer health benefits compared to no MSAs at all (15, 25). This also aligns with the overarching physical activity messaging encouraging even small increments in activity (1, 55, 56).

Beyond saving time, several of the strategies outlined above may simultaneously address other common barriers to MSA engagement. For example, by considering varying equipment availability, we have outlined how MSAs guideline adherence can be achieved in a time-efficient way, even without having additional exercise equipment (e.g., dumbbells) or a gym membership—another important barrier to engaging in MSAs/RT (42, 43). It should be noted, though, that having some equipment (e.g., elastic bands, dumbbells) or access to an exercise facility drastically increases the range of exercises that can be performed and may also make achieving progressive overload easier. That way, more effective programs could be designed and/or variation increased, which—we hypothesize (57)—might help with long-term maintenance of the program. However, the introduced approaches may be a starting point for many people who do not currently meet MSAs guidelines. Additionally, while home-based RT can be a valid form of MSA, starting with some of the exemplary home-based RT sessions described herein may also be a stepping stone toward other forms of RT (e.g., structured, gym-based RT) over time for some individuals. Furthermore, showcasing how different programs may look like that still align with the WHO guideline on MSAs may also enable individuals to pick and choose elements they like and discard elements they do not like, thereby potentially increasing the enjoyability of MSAs—another important starting point for their promotion (42, 43).

### 5.1 Issues with tailoring MSAs prescription to current public health guidelines

While the 2020 WHO guideline on MSAs served as the basis for the present article (1), several unspecified aspects of this guideline—and similar public health guidelines on MSAs—leave considerable room for interpretation, which extends to corresponding exercise prescription. A fundamental issue pertains to the fact that it remains unclear which specific activities should be classified as MSAs (58). In this Perspective, we focused on RT as the core MSA. However, there is no consensus as to whether exercise and non-exercise activities beyond RT count as MSAs (35, 58–60). It would be highly beneficial to know which other activities could be prescribed as MSAs in practice. Indeed, the decision which activities are counted as MSAs also strongly impacts results on MSAs guideline adherence (35, 58), for example. Similarly, greater specificity regarding other MSA prescription variables would be important to consider in future MSA guidelines: (1) It is not specified which muscular adaptation is the primary goal of engaging in MSAs, e.g., whether persons should prioritize muscle hypertrophy, maximal strength, power, or local muscular endurance (11). While there is overlap in the program design for these different outcomes, variables like the loading schemes or the set structure (8, 61, 62) may vary in RT programs that prioritize one outcome over the others. Additionally, non-RT activities that might count as MSAs may also differ in their impact on these distinct muscular adaptations. Thus, knowing which muscular adaptation is the primary goal of engaging in MSAs could also help shape future MSAs guidelines in terms of recommendations regarding additional variables like RT volume (e.g., number of RT sets per muscle group per week) or recommendations on potential non-RT MSAs. (2) Additional room for interpretation exists because it is not specified which definition of intensity the recommendation to perform MSAs at moderate or greater intensity refers to, e.g., whether relative intensity expressed as the percentage of the 1-repetition maximum or intensity of effort (i.e., proximity to muscular failure) should be used for prescribing MSAs. (3) The guideline on MSAs frequency does not specify if all major muscle groups should be trained in all sessions, or only some sessions. In other words, the recommended frequency per muscle group or exercise is not clear. The rather unspecific nature of these recommendations has major implications for programming, e.g., pertaining to the use of full-body vs. split routines.

### 5.2 Implications and directions for future research

While the relatively unspecific nature of current public health guidelines on MSAs makes their translation into concrete, actionable programs challenging for practitioners, public health communicators, and individuals alike, herein we have attempted to provide practical approaches. Based on the available evidence, it would be expected that the example RT programs provided would induce measurable neuromuscular adaptations (e.g., improvements in muscle strength and mass), which is of particular importance in an aging world due to age-related declines in muscle mass, strength, and power (12). We encourage researchers to study the efficacy and effectiveness of specific time-efficient RT prescriptions (e.g., those proposed herein or similar), so that future public health and clinical practice guidelines could include them and individuals could be provided with very specific recommendations.

## 6 Conclusion

MSAs like RT have unique health benefits, but most people in the general population do not perform MSAs in line with public health guidelines. The present Perspective described how – by bridging the gap between public health and sports/exercise science research – time-saving strategies can be used to design time-efficient RT programs aimed at meeting the WHO guideline on MSAs with a weekly time commitment of only about 40 to 60 min. Some of the outlined strategies additionally address other common barriers to MSA engagement, including a lack of equipment and the need for beginner-friendly approaches, further underscoring their practical relevance.

## Data Availability

The original contributions presented in the study are included in the article/supplementary material, further inquiries can be directed to the corresponding author.
